# Definition of the zebrafish genome using flow cytometry and cytogenetic mapping

**DOI:** 10.1186/1471-2164-8-195

**Published:** 2007-06-27

**Authors:** Jennifer L Freeman, Adeola Adeniyi, Ruby Banerjee, Stephanie Dallaire, Sean F Maguire, Jianxiang Chi, Bee Ling Ng, Cinthya Zepeda, Carol E Scott, Sean Humphray, Jane Rogers, Yi Zhou, Leonard I Zon, Nigel P Carter, Fengtang Yang, Charles Lee

**Affiliations:** 1 Department of Pathology, Brigham and Women's Hospital, Boston, Massachusetts 02115, USA; 2 Harvard Medical School, Boston, Massachusetts 02115, USA; 3 Wellcome Trust Sanger Institute, Wellcome Trust Genome Campus, Hinxton, Cambridge, CB10 1SA, UK; 4 Centro de Ciencias Genomicas, Universidad Nacional Autónoma de México, Ap. Postal 565-A, Cuernavaca, Morelos, Mexico; 5 Howard Hughes Medical Institute and Division of Hematology/Oncology, Children's Hospital and Dana-Farber Cancer Institute, Harvard Medical School, Boston, Massachusetts 02115, USA

## Abstract

**Background:**

The zebrafish (*Danio rerio*) is an important vertebrate model organism system for biomedical research. The syntenic conservation between the zebrafish and human genome allows one to investigate the function of human genes using the zebrafish model. To facilitate analysis of the zebrafish genome, genetic maps have been constructed and sequence annotation of a reference zebrafish genome is ongoing. However, the duplicative nature of teleost genomes, including the zebrafish, complicates accurate assembly and annotation of a representative genome sequence. Cytogenetic approaches provide "anchors" that can be integrated with accumulating genomic data.

**Results:**

Here, we cytogenetically define the zebrafish genome by first estimating the size of each linkage group (LG) chromosome using flow cytometry, followed by the cytogenetic mapping of 575 bacterial artificial chromosome (BAC) clones onto metaphase chromosomes. Of the 575 BAC clones, 544 clones localized to apparently unique chromosomal locations. 93.8% of these clones were assigned to a specific LG chromosome location using fluorescence *in situ *hybridization (FISH) and compared to the LG chromosome assignment reported in the zebrafish genome databases. Thirty-one BAC clones localized to multiple chromosomal locations in several different hybridization patterns. From these data, a refined second generation probe panel for each LG chromosome was also constructed.

**Conclusion:**

The chromosomal mapping of the 575 large-insert DNA clones allows for these clones to be integrated into existing zebrafish mapping data. An accurately annotated zebrafish reference genome serves as a valuable resource for investigating the molecular basis of human diseases using zebrafish mutant models.

## Background

The zebrafish (*Danio rerio*) has long been appreciated as a unique model system for vertebrate genetics and developmental biology. The zebrafish genome is about half the size of most mammalian genomes [1] containing some 4.6 pg of DNA [2] distributed across 25 pairs of chromosomes (2*n *= 50) [3]. Initial comparisons of zebrafish and mammalian gene maps have revealed extensive conservation of syntenic chromosome regions among vertebrates [4-10]. Hence, through the use of zebrafish mutants and a well annotated zebrafish reference genome, genetic mechanisms that are conserved among all vertebrates, can be investigated.

Since the launch of the Trans-NIH Genome Initiative in 1997 [11], excellent genetic and genome resources have been established to facilitate the genetic analysis of zebrafish. The first genetic linkage map was constructed using haploid genetics with random amplified polymorphic DNA (RAPD) markers [12,13]. Several meiotic panels (e.g., the Boston MGH Cross, GAT, Heat Shock, and Mother of Pearl) and radiation hybrid (RH) panels (e.g., T51 and LN54) have been generated [9,14-25]. Most recently, the Wellcome Trust Sanger Institute (Hinxton, United Kingdom) released the sixth zebrafish genome assembly (Zv6), comprising of 1.6 Gb [26]. This assembly was generated from 7,615 BAC clones, which were fingerprinted and then arranged into contigs by overlap analysis to generate a physical map. DNA sequence data from whole genome shotgun contigs were then used to fill gaps. While more high-throughput methods for DNA sequencing are now emerging (e.g., the 454 sequencing platform – which can sequence 25 million bases in about four hours [27]), a major limitation of many of these technologies is the provision of relatively short DNA sequences (about 100–200 bases per read), making such sequencing technologies problematic with the assembly of repeated DNA elements [28].

Cytogenetic methodologies provide a complementary mapping approach for assembly of reference genomes. In this study, we have employed two cytogenetic approaches to define the zebrafish genome: flow karyotype analysis and chromosomal mapping by fluorescence *in situ *hybridization (FISH). In flow karyotyping, each chromosome is labeled directly with two DNA-binding fluorochromes (i.e., Hoechst 33258 and Chromomycin A3) and then analyzed on a flow cytometer. The fluorescence from the two DNA-binding dyes enables different chromosome types to be identified by both DNA content and base-pair ratio, providing a means to estimate the relative size of each zebrafish chromosome. In FISH, cloned DNA sequences are hybridized to their complementary sequences and visually mapped onto metaphase chromosomes. In this study, we have unequivocally mapped 575 large-insert DNA clones onto zebrafish metaphase chromosomes using multi-color FISH methods. These clones can be used as anchors for annotation of the zebrafish reference genome and provide independent validation of the sequence map, in a fashion similar to how cytogenetic data from the human genome was integrated into the human DNA sequence map [29]. These data reinforce the use of cytogenetic methodologies for the assembly of complex genomes.

## Results

### Estimation of chromosome sizes based on flow cytometry

**Figure 1:**
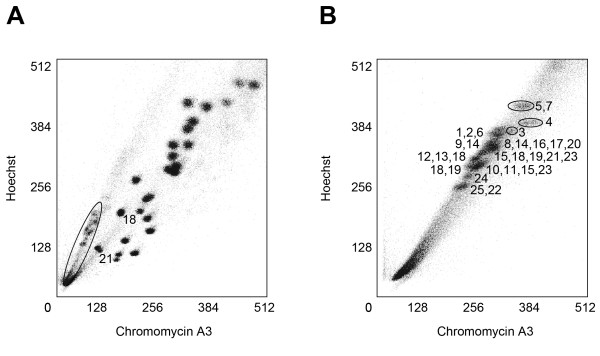
**Estimation of the size of the 25 zebrafish LG chromosome pairs using flow cytometry**. **(A) **A flow karyogram depicting the mixed chromosome preparation of human and zebrafish chromosomes. Flow-peaks contained in the oval represent the zebrafish chromosomes (see B for the chromosomal composition of each peak). The largest zebrafish LG chromosomes (5 and 7) were slightly larger than human chromosome 18 (76.12 Mb) and the smallest zebrafish LG chromosomes (22 and 25) were similar to the size of human chromosome 21 (46.94 Mb). Thus, human chromosomes 18 and 21 were chosen as references (labeled in the karyogram). **(B) **A flow karyogram of the zebrafish chromosome preparation. LG chromosomes ranged in size from 42.2 to 77.9 Mb and the LG chromosomes composing each peak are denoted. The similarity of size resulted in only LG chromosomes 3, 4, and 24 separating into distinct peaks. The remaining nine peaks contained 2 to 5 chromosomes.

Zebrafish chromosomes were analyzed by flow cytometry and the bivariate flow karyotypes obtained for the mixed human-zebrafish and zebrafish chromosome preparations are shown in Figures 1A and 1B, respectively. In contrast to the human chromosomes that are generally well resolved, with the exception of human chromosomes 9–12, the 25 pairs of zebrafish chromosomes were only resolved into approximately 12 peaks. Chromosomes were flow sorted from each peak and after amplification by DOP-PCR, hybridized together with chromosome-specific markers [30] to identify the chromosomal composition of each peak (Figure 1B). It should be noted that since there is an absence of a consensus karyotype, the zebrafish community has chosen to name each chromosome based upon syntenic markers to a given linkage group (LG). For example, markers on LG 1 have been cytogenetically mapped to a particular chromosome, which is now designated as LG chromosome 1. Three peaks each contained one chromosome (LG chromosomes 3, 4, 24), while the remaining nine peaks each contained 2 to 5 chromosomes (LG chromosomes 5+7, 1+2+6, 9+14, 8+14+16+17+20, 12+13+18, 15+18+19+21+23, 18+19, 10+11+15+23, 22+25) due to the similarity of chromosomal size and AT/GC ratio of the non-homologous chromosomes in each peak. The largest chromosomes (i.e., LG chromosomes 5 and 7) are of similar size to human chromosome 18, while the smallest chromosomes (i.e., LG chromosomes 22 and 25) are slightly smaller than human chromosome 21. Using human chromosomes 18 and 21 as references, we have estimated the sizes of all 25 zebrafish chromosomes from the flow karyotype (Table 1).

**Table 1:** BAC clones cytogenetically mapped by FISH to a unique chromosomal location

**LG chromosome**	**Chromosome size determined by flow cytometry (Mb)**	**Number of BAC clones uniquely mapped by FISH**	Percent coverage within a chromosome*
1	64.9	21	5.7
2	64.9	37	10.0
3	68.1	28	7.2
4	73.6	51	12.1
5	77.9	17	3.8
6	64.9	14	3.8
7	77.9	17	3.8
8	60.6	17	4.9
9	56.8	17	5.2
10	51.7	19	6.4
11	51.7	23	7.8
12	52.8	16	5.3
13	52.8	28	9.3
14	58.7	12	3.6
15	52.7	15	5.0
16	60.6	22	6.4
17	60.6	15	4.3
18	51.9	18	6.1
19	51.5	20	6.8
20	60.6	31	9.0
21	53.6	11	3.6
22	43.3	27	10.9
23	52.7	10	3.3
24	46.5	13	4.9
25	42.2	11	4.6

Total	1453.5	510	6.1% of genome

*BAC clone average insert size equal to ~175 kb

### Chromosomal mapping of 575 BAC clones by FISH

A total of 670 BAC clones were processed for cytogenetic mapping in the zebrafish genome. The LG chromosome for which each BAC clone was predicted to reside, was determined by searching four available zebrafish databases (i.e., (1) the University of California – Santa Cruz zebrafish genome browser (UCSC) [31], (2) the Ensembl zebrafish genome browser [32], (3) Vega, a zebrafish genome annotation tool [33], and (4) the Zebrafish Genome Fingerprinting Project Clone database (WebFPC) [34]). Mapping information was available in at least one of the databases for most all the clones, while there was almost always some discrepancy among the four databases (Table 2; Additional file 1; Additional file 2). Each BAC probe was labeled with a fluorochrome (e.g., spectrum orange or spectrum green) and combined with a near-centromeric or near-telomeric probe (in a second color) from the same LG chromosome, as defined by our first generation zebrafish BAC clone probe panel [30]. BAC clones were initially mapped onto metaphase chromosomes from an established and stable fibroblast cell line from an AB strain zebrafish and also confirmed on metaphase chromosomes from wild-type AB and Tu embryos.

**Table 2:** 510 uniquely mapped BAC clones compared to LG chromosome assignment in four zebrafish genome databases

	No. of clones*	**Percent of clones**
UCSC genome browser		
Not in database	74	14.5
Present in database	436	85.5
Agree	331	75.9
Disagree	78	17.9
Mapped to "U" ^†^	27	6.2
Ensembl (Zv6-Release 40) ^‡^		
Not in database	262	51.4
Present in database	248	48.6
Agree	215	86.7
Disagree	29	11.7
Mapped to "U"	4	1.6
Sanger WebFPC (August 2006)		
Not in database	123	24.1
Present in database	387	75.9
Agree	358	92.5
Disagree	29	7.5
Mapped to "U"	0	0.0
Vega (version 20)		
Not in database	211	41.4
Present in database	299	58.6
Agree	255	85.3
Disagree	26	8.7
Mapped to "U"	18	6.0

*Total number of clones based upon the 510 BAC clones assigned to a unique LG chromosome by FISH mapping

^†^Mapped unambiguously to an unknown chromosome (chromosome U)

^‡^Zv6 (version 6-Release 40) of Ensembl does not contain 233 Danio Key (zK) BAC clones which were uniquely assigned to a specific LG chromosome in this study

Data obtained as of August 2006

**Figure 2:**
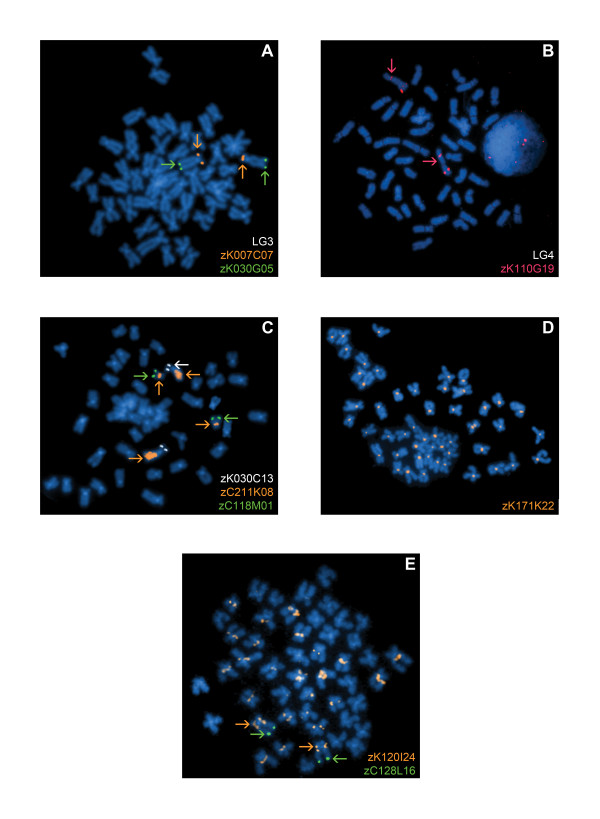
**The processing of 670 BAC clones for cytogenetic mapping in the zebrafish genome by FISH**. **(A) **BAC clone zK030G05 (labeled in green, denoted by green arrows) is observed to be syntenic with a previously mapped BAC clone probe, zK007C07, which localizes to the short arm (p arm) of LG chromosome 3, (labeled in orange, denoted by orange arrows). **(B) **One BAC clone, zK110G19 (labeled in red), was observed to have two signals on the same chromosome, LG chromosome 4. The primary signal is on 4p with a secondary signal in the heterochromatic region of 4q. LG chromosome 4 is denoted by red arrows. **(C) **Seventeen clones had signals on two non-homologous chromosomes. For example, zC211K08 (labeled in orange) localized to the p arm of LG chromosome 22 and to the q arm heterochromatic region of LG chromosome 4 (denoted by orange arrows). The near-telomeric marker for the q arm of LG chromosome 22, zC118M01, is labeled in green (denoted by green arrows) and the near-telomeric marker for the p arm of LG chromosome 4, zK030C13, is labeled in white (denoted by white arrows). **(D) **Five BAC clones were pan-centromeric, such as zK171K22 (labeled in orange). **(E) **Five BAC clones were peri-centromeric. For example, zK120I24 (labeled in orange) localized to the p arm and near the centromere of LG chromosome 7 (denoted by orange arrows), the p arm and near the centromere of an unknown chromosome, and near the centromere of multiple chromosomes. The near-telomeric marker for the q arm of LG chromosome 7, zC128L16, is labeled in green (denoted by green arrows).

Of the 670 BAC clones that were processed for FISH mapping, a total of 544 BAC clones localized to apparently unique chromosomal locations on metaphase chromosome spreads. 93.8% (510 clones) of these clones, approximately 6.1% of the zebrafish genome (Table 1), were assigned to a specific LG chromosome (Additional file 1; e.g., Figure 2A). Of the 510 BAC clones assigned to a unique LG chromosome by FISH mapping, Ensembl and Vega did not have mapping information on a substantial number of these clones (51.4% and 41.4%, respectively; Table 2). Ensembl Zv6 (version 6-release 40, August 2006) did not contain any clones from the Danio Key (zK) library investigated in this study (233 of the 510 clones). When evaluating only those clones present in the databases, LG chromosome assignments agreed for 75.9% to 92.5% of the clones and disagreed for 7.5% to 17.9% of the clones, dependent upon the database (Table 2). A common discrepancy between the predicted LG chromosome location and the assigned LG chromosome from FISH mapping, occurred for BAC clones which were found to chromosomally localize to the heterochromatic region on the long arm (q arm) of LG chromosome 4. 40.0% (10 out of the 25 clones) of the clones that uniquely FISH mapped to the heterochromatic region of LG chromosome 4 were assigned to LG chromosome 14 by at least one of the databases (Additional file 1).

Thirty-one clones (4.6%) mapped cytogenetically to multiple chromosomal locations (Additional file 3). One of the clones had two signals on the same chromosome (Figure 2B), 17 clones had hybridization signals on two different, non-homologous chromosomes (e.g., Figure 2C), 1 clone had signals on three different, non-homologous chromosomes (data not shown), and 2 clones had signals on four different, non-homologous chromosomes (data not shown). Five BAC clones were pan-centromeric (i.e., hybridized near the centromere of all chromosomes; e.g., Figure 2D) and five BAC clones were peri-centromeric (i.e., hybridized near the centromere of multiple chromosomes; e.g., Figure 2E).

**Figure 3:**
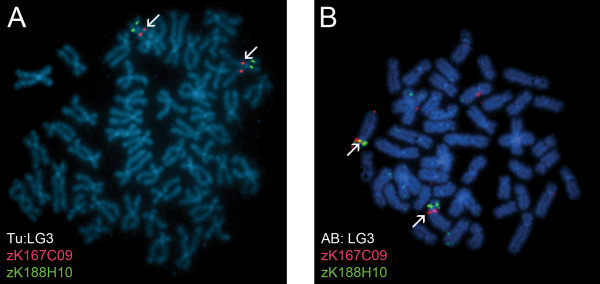
**A chromosome inversion variant observed between the Tu strain and the AB strain**. **(A) **In metaphase preparations from Tu embryos, BAC clone zK167C09 (labeled in red) was observed to have only homozygous signals near the centromere of LG chromosome 3q. zK188H10, which consistently localized near the middle of LG chromosome 3p, is labeled in green. **(B) **Three hybridization patterns were observed for zK167C09 in metaphase preparations from AB embryos including homozygous signals near the centromere of LG chromosome 3q, homozygous signals located medially on LG chromosome 3p, and a heterozygous state. The heterozygous pattern is depicted in this image with zK167C09 labeled in red and zK188H10, which consistently hybridized medially on LG chromosome 3p, labeled in green. White arrows denote LG chromosome 3 in **(A) **and **(B)**.

One consistent discrepancy was observed in the mapping results between the two zebrafish strains analyzed (Tu and AB). The BAC clone zK167C09 consistently localized near the centromere of the long arm of LG chromosome 3 in the Tu strain (Figure 3A), but had three different hybridization patterns in the metaphase preparations from AB embryos. In certain embryos, a homozygous state was observed with zK167C09 localizing to the long arm peri-centromeric region of LG chromosome 3, as seen with the Tu strain. Other AB embryos exhibited a heterozygous state with zK167C09 localizing near the centromere of one homolog of LG chromosome 3, while hybridization on the other homolog was in the middle of the short arm (Figure 3B). Other AB embryos revealed zK167C09 hybridization signals in the middle of the short arm of both homologs of LG chromosome 3. This result is consistent with the existence of a polymorphic peri-centromeric chromosomal inversion in the zebrafish AB strain.

A total of 95 BAC clones (14.2%) failed to produce distinct and reproducible FISH signals, even after multiple hybridization attempts. These clones may be shorter than the average BAC clone or could contain substantial amounts of highly repetitive elements that are largely out competed by unlabeled C_o_t1 DNA during the FISH procedure.

### Development of a second generation probe panel

**Figure 4:**
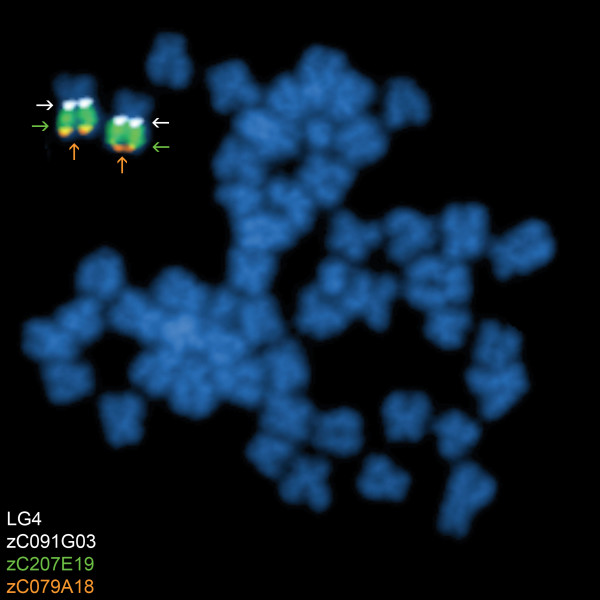
**Identification of a near-telomeric marker for the long arm of LG chromosome 4**. A large portion of the q arm of LG chromosome 4 consists of a heterochromatic region, which made finding a near-telomeric marker probe difficult for this chromosome arm. zC079A18 (labeled in orange) was established as a near-telomeric marker probe for the q arm of LG chromosome 4 (denoted by orange arrows). Also shown is the near-centromeric marker for LG chromosome 4, zC091G03, (labeled in white, denoted by white arrows) and BAC clone zC207E19, which localizes to the heterochromatic region of the long arm of LG chromosome 4 (labeled in green, denoted by green arrows).

While carrying out the FISH mapping assessments, particular attention was given to BAC clones that localized near the centromere or the telomeres of each LG chromosome. Subsequently, an improved second generation probe panel has now been established using BAC clones that were visually closer to these chromosomal domains compared to corresponding clones in the first generation probe panel. This second generation probe panel has substituted 31 of the 75 BAC clones (41.3%; Table 3) and also includes a telomeric clone for the long arm of LG chromosome 4 (Figure 4), which was not previously available.

**Table 3:** Second generation near-centromere and near-telomere zebrafish BAC clone probe panel*

**LG chromosome**	**Near-telomere short arm (p)**	**Near-centromere**	**Near-telomere long arm (q)**
1	zC093G23	zC022O06	zC141F18
2	zK014G06	***zK127K09***	zC009D09
3	zK007C07	zC115J06	***zK030G05***
4	zK030C13	***zC091G03***	***zC079A18***
5	zC087E10	zK007B18	zC150K20
6	***zK023D07***	***zC122J16***	***zK166J19***
7	zK009M06	zK014N10	zC128L16
8	zC069A12	zC103G04	***zK149F22***
9	zC115B08	***zC212N06***	zC012N08
10	zC128P08	***zC136O04***	zC022E09
11	***zC159E12***	***zK014H17***	zC115I06
12	zC121C04	zK022H21	zC086E02
13	zK006L12	***zK016I06***	***zC113I09***
14	zC117N19	zC117E17	zC125N22
15	zC055C01	zC125H09	***zK151P21***
16	***zC119D19***	***zC213B07***	***zK246M23***
17	zK013L17	zK006P15	***zC042B22***
18	***zC241L24***	zK005J13	zK014D24
19	***zK089B17***	zC132A16	***zK201G07***
20	***zK077B17***	***zK033I22***	***zC153J24***
21	zC122A16	zC065O02	zK014M09
22	zK002J07	***zC206A19***	***zC118M01***
23	zC041B11	zC051C19	***zC214H13***
24	***zK018N12***	zK001A04	***zK124I03***
25	zC096F02	***zK044K01***	zC087L10

*Second generation BAC clones are in bold and italics

## Discussion

Following analysis of the initial zebrafish gene maps, it has been suggested that during the course of evolution, the zebrafish and human lineages shared two rounds of whole genome duplication with a third whole genome duplication event occurring before the last teleost radiation [10,35]. The duplicated genome of the zebrafish has contributed to the increased difficulty in constructing an accurate genetic map for this organism, in comparison to the mouse and human. In addition, similarity in the size, morphology, and banding pattern of the zebrafish chromosomes has resulted in at least 12 different published zebrafish karyotypes [reviewed in [36]]. Phillips et al. [37] used a combination of relative size and chromosome arm ratios to estimate the sizes of each zebrafish chromosome. When comparing the chromosome size estimates from our flow cytometry data to the size estimates reported by Phillips et al. [37], the four largest LG chromosomes (i.e., LG chromosomes 5, 7, 4, and 3) and the three smallest LG chromosomes (i.e., LG chromosomes 22, 24, and 25) are in agreement. However, the ranking of sizes of most other LG chromosomes are discrepant and should be investigated further by other independent studies.

Cytogenetic mapping is a complementary approach to other mapping strategies (e.g., genetic and radiation hybrid mapping) and can be used to confirm existing framework maps. Using fluorescence *in situ *hybridization (FISH), 510 BAC clones were assigned to unique LG chromosome locations in this study. This suggests that if there is widespread duplication in the zebrafish genome, a large proportion of these appear to be syntenic and localized (e.g., tandem duplications). FISH mapping data was over 85% concordant for three of the four zebrafish genome databases (i.e., Ensembl, Vega, and WebFPC) with 75.9% concordance for the UCSC Genome Browser, when evaluating the BAC clones present in these databases (Table 2). Discrepancies between the FISH mapping data and the LG chromosome assignment by the zebrafish genome databases may be attributed to cross-contamination of the clones, clone tracking inconsistencies, incorrect assignment by previous mapping efforts, or inconsistencies among the four zebrafish genome databases. From the comparisons, it became evident that at the time of analysis, there is some degree of discrepancy among all four of the zebrafish genome databases. In addition, the Ensembl Zv6 (version6-release 40, August 2006) used in this analysis did not contain any of the BAC clones of the zK library. Of the 510 BAC clones that were assigned to specific LG chromosomes by FISH, only 3 clones did not have mapping information in any of the databases. In addition for 53 of the 507 clones with database mapping information, none of the databases had the same chromosomal assignment as was assigned by FISH (Additional file 1).

A small percentage of the clones were observed to hybridize to multiple locations within the zebrafish genome (Additional file 3). One clone contained duplicated regions within the same chromosome, while the remaining thirty clones hybridized to more than one chromosome. Many of the BAC clones that hybridized to multiple chromosomal locations localized near the centromeres, providing further evidence for the lack of chromosome-specific repetitive DNA sequences near these regions. These clones provide clues to duplications and exchanges that have occurred within and between chromosomes. Further investigation is needed to determine the functional significance of these duplications.

Some BAC clones yielded weak or no fluorescence signals. The lack of signals for these clones may be due to poor quality of probe DNA, contamination with non-zebrafish DNA, loss of insert DNA or poor hybridization due to factors such as increased repetitive DNA elements within a clone.

One particularly fascinating observation was a peri-centromeric inversion on LG chromosome 3 (Figure 3). Interestingly, this heteromorphism was seen only in the AB strain and not in Tu embryos. This finding suggests that there are strain-specific chromosomal differences in the zebrafish. Large chromosomal rearrangements (e.g., inversions) are important for evolution and are suspected to play a role in speciation. Studies have suggested that inversions can influence patterns of nucleotide diversity between diverging taxa [38] and encourage speciation by suppressing recombination in heterozygotes [39]. Chromosomal inversions can also play a role in fertility. For example, peri-centromeric inversion carriers may have a reduction in fertility due to the production of chromosomally unbalanced gametes. In a recent study, it has also been suggested that chromosomal inversions may be beneficial. Carriers for an inversion on human chromosome 17 were reported to have increased fertility rates in comparison to non-carriers [40].

Chromosomal mapping of zebrafish BAC clones by FISH in this study resulted in a second generation near-centromere and near-telomere BAC clone probe panel, which includes 41.3% new clones. These new BAC clones are visually mapped closer to the centromere or telomeres of each LG chromosome than those in the original probe panel. Furthermore, a near-telomeric BAC clone was identified at the long arm terminus of LG chromosome 4 (Figure 4). The long arm of LG chromosome 4 (i.e., chromosome 3 as denoted by the karyotype of Gornung et al. [41] and Phillips et al. [37]) has been previously shown to contain a substantial amount of constitutive heterochromatin [36]. Regions of constitutive heterochromatin often consist of highly repetitive DNA and are largely transcriptionally silent. The heterochromatic region of LG chromosome 4 is late replicating [42], stains darker than the short arm by C-banding [41], and is reported to also harbor 5S rDNA [43,44]. In the current study, 25 BAC clones mapped uniquely to the heterochromatic region of LG chromsome 4 (e.g., Figure 4). Moreover, five BAC clones that mapped to multiple chromosome locations included this heterochromatic region on LG chromosome 4 (Additional file 3; Figure 2C). This would suggest a complex genome structure of multiple repetitive DNA families in this region on LG chromosome 4, some of which are shared sequences at other chromosomal regions.

## Conclusion

We have used flow cytometry and chromosomal mapping to develop a cytogenetic map of the zebrafish genome. These data can be integrated with genetic and radiation hybrid mapping data to produce a refined sequence map and to confirm the existing genomic framework of the zebrafish. A refined zebrafish genome serves as a valuable resource for genetic research that utilizes this model organism for understanding of the molecular basis of human diseases. For example, the mapped clones can be used to narrow the search for candidate genes, which may be altered in zebrafish mutant models using FISH assays. This strategy was recently used to identify a loss of one allele of the separase gene, when a probe containing the separase gene locus was hybridized upon epithelial tumor specimen, indicating that a loss of one copy of separase is sufficient to promote tumorigenesis in the zebrafish [45]. The cytogenetically mapped clones can also be used in the development of a zebrafish array comparative genomic hybridization platform for use in identifying genomic imbalances in developmental and disease zebrafish models. Integrating these BAC clones with the zebrafish reference sequence will allow investigators to directly determine the genomic regions that are gained or lost. Overall these data contribute towards the ultimate defintion of a reference zebrafish genome and provide insight into the evolution of vertebrate chromosomes.

## Methods

### Chromosome preparation

#### AB fibroblast cell line

The primary fibroblast cell line was established from approximately 100 embryos of the AB strain fishes, and grown in Advanced DMEM (Gibco) medium, supplemented with 15% fetal bovine serum (FBS, Gibco) and antibiotics (Penicillin and Streptomycin, Sigma). At about 70% confluency, cells were treated with 0.1 μg/ml colcemid, then placed in a hypotonic solution (0.067 M potassium chloride) at room temperature for 15 min., and then fixed in ice-cold Carnoy's methanol:glacial acetic acid (3:1) fixative. Chromosomes were dropped onto slides and allowed to dry overnight at 37°C.

#### AB and Tuebingen embryos

Methods are adapted from Lee and Smith [30]. At 23 hours post-fertilization, 100 embryos were treated with colchicine (4 mg/ml) for 6 hours. Embryos were then dechorionated by pronase treatment [46] and lightly homogenized. Ice-cold 0.9× phosphate buffered saline (PBS; Sigma, St. Louis, MO), 10% fetal bovine serum (BioWhittaker, Walkersville, MD) was added and embryos filtered subsequently through a 100 μm and 50 μm mesh. Embryos were then centrifuged at 250 rcf for 10 min. at 4°C. Supernatant was decanted and embryos were treated for 25 min. at room temperature in a hypotonic solution (1.1% sodium citrate, 4 mg/ml colchicine). Cells were centrifuged at 450 rcf for 10 min. at 4°C and ice-cold Carnoy's methanol: glacial acetic acid fixative (3:1) was added. Centrifugation was repeated and fresh fixative added. Chromosomes were then dropped onto slides and allowed to dry overnight at 37°C.

### Characterization of zebrafish chromosome sizes by flow cytometry

Chromosomes were prepared from human lymphoblastoid HRC159 cells and from the zebrafish AB strain fibroblast cell line. Twenty-four hours after subculturing, the cell cultures were treated with colcemid (0.1 μg/ml) for 6 hours. Chromosomes were prepared by using modifications of a polyamine isolation method [47]. For the HRC159 cell line, 50 ml of blocked cell culture was centrifuged at 289 rcf for 5 min. and the cell pellet was resuspended in 5 ml of hypotonic solution (75 mM KCl, 10 mM MgSO_4_, 0.2 mM spermine, 0.5 mM spermidine, pH 8.0) and incubated at room temperature for 10 min. For the zebrafish cell lines, the supernatant from six 150 cm^2 ^flasks after mitotic shake-off was collected and centrifuged at 289 rcf for 5 min. The cell suspensions were pooled together into one tube after resuspending the cell pellets in hypotonic solution (5 ml). The suspension of swollen cells was centrifuged at 289 rcf for 5 min. The cell pellets of HRC159 cells and zebrafish were each resuspended in 3 ml and 1 ml of ice cold polyamine isolation buffer (PAB, containing 15 mM Tris, 2 mM EDTA, 0.5 mM EGTA, 80 mM KCl, 3 mM Dithiothreitol, 0.25% Triton X-100, 0.2 mM Spermine, 0.5 mM spermidine, pH 7.5), respectively, and vortexed for 20 sec. For the zebrafish cells, this was followed by forcing the suspensions twice through a 21 gauge needle (Becton Dickinson, Microlance™ 3) attached to a 2 ml syringe (Becton Dickinson, Plastipak™).

All chromosomes suspensions were then briefly centrifuged (201 g, 2 min.) and the supernatant was filtered through a 20 μm mesh filter (Celltrics, Partec). An aliquot of zebrafish chromosome suspension was mixed with HRC159 and stained for at least 2 hours with 5 μg/ml of Hoechst (Sigma) and 40 μg/ml of Chromomycin A3 (Sigma). 10 mM MgSO_4_, 10 mM sodium citrate and 25 mM sodium sulphite was added to the stained preparation one hour before flow analysis. The stained chromosome suspension was analyzed on a flow cytometer (MoFlo^®^, DAKO) as described elsewhere [47]. A total of 100,000 events were acquired for each cell line at a rate of 1000 events per second. The flow karyotype of the stained chromosomes was displayed as a bivariate flow karyogram of Hoechst versus Chromomycin fluorescence. Data collected from the experiments were analysed using Summit V3.1 (analysis software from DAKO) and the size of zebrafish chromosomes in mega-basepairs of DNA was estimated based on a method described previously [48-51] using the distribution of human chromosomes as the reference. Briefly, this method uses the projection of each flow peak in the flow karyotype onto the 'DNA line' which passes chromosome 4 and the origin. The distance from the origin to the point of projection on the 'DNA line' is proportional to the DNA content of the chromosome. Since the largest LG chromosomes and the smallest LG chromosomes of zebrafish are close to the human chromosomes 18 and 21, respectively, the sizes of human chromosomes 18 (76.12 Mb) and 21 (46.94 Mb) were used as the references.

### Bacterial Artificial Chromosome (BAC) clone DNA preparation

Clones from the CHORI-211 (zC), Danio Key (zK), Danio Key Pilot (zKp), RPCI-71 (bZ), and CHORI-73 (zH) zebrafish BAC DNA libraries were streaked on LB-Agar plates containing the appropriate antibiotic and grown overnight at 37°C. A single colony was then inoculated in TB media containing the appropriate antibiotic and placed in a shaking incubator for 16 hours at 37°C. DNA was isolated by the Qiagen Plasmid Midi Kit protocol (Valencia, CA), amplified using the Repli-G Midi protocol (Qiagen, Valencia, CA) and submitted for sequencing (Additional file 4). Alternatively, the DNA was prepared using the BAC Phase-Prep kit (Sigma).

### Fluorescence *in situ *Hybridization (FISH)

BAC DNA probe was labeled by adding 1 μg of DNA, 5 μl of 10× translation buffer (500 mM Tris-HCl, 100 mM MgSO_4_, 1 mM dithiothreitol), 10 μl dNTP mix (0.1 mM dATP, 0.1 mM dCTP, 0.1 mM dGTP), 5 μl of 0.1 mM dTTP, 10 μl of nick translation enzyme (5 units DNA polymerase I, 0.1 units DNAse I, 50 mM Tris-HCl, 10 mM MgSO_4_, 0.1 mM dithiothreitol, 0.5 mg/ml bovine serum albumin), 2.5 μl of 0.2 mM Spectrum Orange dUTP (Abbott Vysis, Des Plaines, IL) or Spectrum Green dUTP (Abbott Vysis, Des Plaines, IL), and water to a volume of 50 μl. The mixture was incubated at 15°C for 11 hours and the DNA cleaned using a Sephadex-G50 column (Zymo Research, Orange, CA). Alternatively, the BAC DNA was labeled with biotin- and digoxigenin-dUTP by nick translation using the Roche kits following the protocols supplied by the manufacturer. Twenty-five micrograms of C_o_t1 DNA was added and DNA lyophilized. DNA pellets were resuspended in hybridization buffer (50% formamide, 2× SSC, 10% Dextran Sulfate).

For hybridization, slides were pretreated for 15 min. in ice-cold Carnoy's methanol:glacial acetic acid (3:1) fixative, washed briefly in PBS, digested at 37°C in Protease II for 5 min., and then dehydrated in an ethanol series (70%, 90%, 100%). Following slide pretreatment, 5 μl (0.25 μg) of the DNA probe was added onto the slides under a coverslip and sealed with rubber cement. The DNA probe was denatured for 3 min. at 70°C and then incubated in a humidified chamber in the dark at 37°C for at least 24 hours.

Post-hybridization washes comprised of 50% formamide, 2× SSC at 45°C, 2× SSC at 45°C, and then 4× SSC, 0.05% Tween 20 at 37°C. Slides were counterstained in DAPI (Vector Laboratories, Burlingame, CA), and then analyzed by fluorescence microscopy on an Olympus BX-51 fluorescence microscope equipped with narrow band pass filters for Spectrum Orange and Spectrum Green dyes. Images were captured using a Photometrics KAF1400 CCD camera and Applied Imaging Genus Software system. At least 10 metaphase spreads were analyzed per BAC clone.

## Abbreviations

BAC, Bacterial artificial chromosome; FISH, fluorescence *in situ *hybridization; LG, Linkage group; p arm, short chromosome arm; q arm, long chromosome arm; Tu, Tuebingen; zC, CHORI 211 zebrafish BAC library; zK, Danio Key zebrafish BAC library

## Competing interests

The author(s) declare that they have no competing interests.

## Authors' contributions

JLF, FY, and CL designed the experiments. JLF, AA, RB, SD, SFM, JC, BLN, CES, and CZ performed the experiments. SH, JR, YZ, LIZ, and NPC contributed tools and materials. JLF and FY analyzed the data. JLF and CL wrote the paper. All authors read and approved the final manuscript.

## Supplementary Material

Additional file 1 A table listing 510 BAC clones assigned to a unique LG chromosome location by cytogenetic mapping and the predicted LG chromosome location indicated by four zebrafish genome databases (as of August 2006). The 510 BAC clones were assigned to a chromosome, chromosome arm, and region within each chromosome arm as observed with cytogenetic mapping. The current genome assembly position (as of May 2007) for each BAC clone was then integrated with the cytogenetic mapping data to further order the BAC clones within each chromosomal region. It should be noted that LG chromosomes 3, 5, 7, 8, 17, 18, 21, 22, and 25 appear to be inverted. Click here for file

Additional file 2 A table summarizing the concordance of the chromosomal locations assigned by cytogenetic mapping with the most current build of the zebrafish genome assembly (Zv6-as of May 2007). Click here for file

Additional file 3 A table listing the BAC clones observed to hybridize to multiple locations within the zebrafish genome using FISH. Click here for file

Additional file 4 A table summarizing the sequencing status of the 510 BAC clones cytogenetically mapped to unique chromosomal locations in this study. Click here for file
