# Supplementary material for: Definition of the zebrafish genome using flow cytometry and cytogenetic mapping
Source: BMC Genomics. 2007 Jun 27;8:195. doi: 10.1186/1471-2164-8-195 (PMC1925092; doi:10.1186/1471-2164-8-195)
Supplement: Additional file 1 — A table listing 510 BAC clones assigned to a unique LG chromosome location by cytogenetic mapping and the predicted LG chromosome location indicated by four zebrafish genome databases (as of August 2006). The 510 BAC clones were assigned to a chromosome, chromosome arm, and region within each chromosome arm as observed with cytogenetic mapping. The current genome assembly position (as of May 2007) for each BAC clone was then integrated with the cytogenetic mapping data to further order the BAC clones within each chromosomal region. It should be noted that LG chromosomes 3, 5, 7, 8, 17, 18, 21, 22, and 25 appear to be inverted. [file 1471-2164-8-195-S1.pdf]

Additional file 1. A table listing 510 BAC clones assigned to a unique LG chromosome location by cytogenetic mapping and the predicted LG chromosome location indicated by four zebrafish genome databases (as of August 2006). The 510 BAC clones were assigned to a chromosome, chromosome arm, and region within each chromosome arm as observed with cytogenetic mapping. The current genome assembly position (as of May 2007) for each BAC clone was then integrated with the cytogenetic mapping data to further order the BAC clones within each chromosomal region. It should be denoted that LG chromosomes 3, 5, 7, 8, 17, 18, 21, 22, and 25 are inverted.

Chromosomal mapping data								Bioinformatic mapping data		
**BAC clone**	**LG by UCSC genome browser (Zv6)***	**LG by Ensembl (Zv6-release 40)***	**LG by Sanger Web FPC database***	**LG by Vega (v20)***	**LG chromosome assignment by FISH mapping**	**Chromosome arm**	**Relative position along chromosome arm**	**LG by Zv6 (UCSC-May 2007)**	**Chromosome position**	**Mapping done by**
zC093G23	1	1 and 2	1	1	1	p	telomeric	1	5257622-5270959	1 BAC End
zC260P11	18	18	6	6	1	p	telomeric	18	58240764-58390425	2 BAC Ends
zC132G01	NA	1	1	NA	1	p	subtelomeric	1	391825-558853<br>391825-560296	2 BAC Ends
zC172N16	1	1	1	1	1	p	distal	1	3902378-3915652<br>3904118-3917392	1 BAC End
zC281A20	1	1	1	NA	1	p	distal	1	5556971-5696107	2 BAC Ends
zK279K15	NA	NA	1	1	1	p	distal	NA		
zC103I06	1	1	1	1	1	p	medial	1	4068088-4242225	2 BAC Ends
zK020N03	1	NA	1	1	1	p	medial	1	21167682-21306520	2 BAC Ends
zK245P14	1	NA	1	1	1	p	near the centromere	1	21769832-21965463	2 BAC Ends
zC145B13	1	1	1	1	1	p	near the centromere	1	34832677-34985267	2 BAC Ends
zC154P15	1	1	NA	NA	1	p	near the centromere	1	35169267-35182532	1 BAC End
zK025O16	1	NA	1	1	1	p	near the centromere	1	37058682-37292992	2 BAC Ends
zC127F23	1	1	NA	NA	1	p	near the centromere	1	65095752-65096438	T51 RH Map
zK200F03	NA	NA	1	NA	1	p	near the centromere	NA		
zC036C22	1	1	1	NA	1	q	near the centromere	1	47613827-47822613	2 BAC Ends
zC022O06	1	1	1	NA	1	q	near the centromere	1	47741705-47926482	2 BAC Ends
zC154P06	1 and 18	1 and 5	NA	NA	1	q	medial	1 and 18	chr1: 53652167-53665436<br>chr 18:32872567-32885761	1 BAC End<br>1 BAC End
zK242H09	18	NA	18	18	1	q	medial	18	19587551-19600710	1 BAC End
zC196H16	1	1	1	1	1	q	subtelomeric	1	64718027-64947507<br>64718027-64949097	2 BAC Ends
zC141F18	1	1	1	NA	1	q	telomeric	1	65198428-65354961	2 BAC Ends
zC169P22	1	1 and 19	NA	NA	1	q	telomeric	1	65203867-65340024	2 BAC Ends
zK014G06	11	NA	NA	NA	2	p	telomeric	2	2757028-2770178	1 BAC End
zC243A15	NA	NA	2	2	2	p	medial	NA		

zK228I19	NA	NA	2	NA	2	p	near the centromere	NA		
zK063K07	NA	NA	2	2	2	p	near the centromere	NA		
zC220P06	2	2	2	NA	2	q	near the centromere	2	13684547-13911816	2 BAC Ends
zK127K09	2	NA	2	NA	2	q	near the centromere	2	13880704-14051063	2 BAC Ends
zK021K10	2	NA	2	2	2	q	near the centromere	2	14223917-14471712	2 BAC Ends
zC039P05	2	2	2	NA	2	q	near the centromere	2	15278821-15292131<br>61286485-61299784	1 BAC End
zC177G10	2	2, 6, and 7	NA	NA	2	q	near the centromere	2	20406718-20419979	1 BAC End
zK058B18	2	NA	2	2	2	q	near the centromere	NA		
zK074F15	NA	NA	2	2	2	q	near the centromere	NA		
zC117K11	2	2	2	NA	2	q	proximal	2	14901387-15071575	2 BAC Ends
zC057G18	2	2	2	2	2	q	proximal	2	16314770-16489662	2 BAC Ends
zK236A14	2 and 7	NA	2	2	2	q	proximal	2	16970863-16984201	1 BAC End
zK024C02	2	NA	2	2	2	q	proximal	2	20905911-20919059	1 BAC End
zK149I17	2	NA	2	2	2	q	proximal	2	21950811-22184552<br>21972870-22184552	2 BAC Ends
zC236G22	14	14	NA	NA	2	q	proximal	14	28960800-29113654	2 BAC Ends
zC218C08	2	2	NA	2	2	q	medial	2	15999595-16198095	2 BAC Ends
zKp099C04	2	2	2	NA	2	q	medial	2	19623792-19636974<br>21597388-21610643	1 BAC End
zK261L02	2	NA	2	2	2	q	medial	2	19765037-19949626	2 BAC Ends
zK005I22	2	NA	2	2	2	q	medial	2	19947571-20157601	2 BAC Ends
zK218H11	2	NA	2	2	2	q	medial	2	20110190-20328751	2 BAC Ends
zKp093C05	2	2	2	2	2	q	medial	2	20853389-21051983	2 BAC Ends
zK223D07	2	NA	2	2	2	q	medial	2	21948585-21961835<br>22422427-22435677	1 BAC End
zK148A12	2	NA	2	2	2	q	medial	2	24618448-24863658	2 BAC Ends
zC212M21	18	2, 8, and 17	2	2	2	q	medial	2	28271265-28472689	2 BAC Ends
zC106H04	2	2	2	2	2	q	medial	2	28541841-28734733	2 BAC Ends
zK125O18	2	NA	NA	NA	2	q	medial	2	38536675-38726774	2 BAC Ends
zK031B10	2	NA	2	2	2	q	medial	2	40487801-40704156	2 BAC Ends
zC106K21	2	2	2	2	2	q	medial	NA		
zK077B19	2	NA	NA	NA	2	q	medial	NA		
zK216E09	2	NA	2	2	2	q	distal	2	27889759-28082345	2 BAC Ends
zC096I07	15	2	2	2	2	q	distal	2	38914173-38927435	1 BAC End
zC265G22	2	2	2	2	2	q	distal	2	46760071-46773508	1 BAC End
zC057G11	2	2	2	NA	2	q	distal	2	50539656-50552952	1 BAC End
zC137C04	8	2	NA	2	2	q	subtelomeric	2	60326515-60339795	1 BAC End
zC009D09	9	2	2	2	2	q	telomeric	2	55415046-55428047	1 BAC End
zK007C07	15	NA	NA	NA	3	p	telomeric	3	61907519-62124553	2 BAC Ends

zC196B14	3	3	3	3	3	p	subtelomeric	3	59114796-59308455	2 BAC Ends
zK188H10	NA	NA	3	3	3	p	medial	3	58898120-58911362	1 BAC End
zC220A02	3	3 and 7	3	NA	3	p	proximal	3 and 7	chr 3: 6812707-6825787	1 BAC End
									chr 7: 72830073-72843153	1 BAC End
zK167C09	15	NA	3	3	3	p	pericentric inversion in AB to 3q	3	58518799-58671242	2 BAC Ends
zC115J06	3	3	NA	NA	3	q	near the centromere	NA		
zC136A14	3	3	3	3	3	q	proximal	3	49382791-49551157	2 BAC Ends
zK183N06	3 and 6	NA	3	3	3	q	proximal	3	19791040-20000784	2 BAC Ends
zC195G24	22	22	3	3	3	q	proximal	22	26533406-26546695	1 BAC End
zK043P13	NA	NA	3	3	3	q	proximal	NA		
zK108H08	24	NA	3	3	3	q	proximal	NA		
zC256M01	3	3	3	3	3	q	medial	3	29372287-29528745	2 BAC Ends
zC069D20	3	3	3	NA	3	q	medial	3	29318386-29502357	2 BAC Ends
zK271F03	3	NA	3	3	3	q	medial	3	29114886-29128092	1 BAC End
zK077J09	7 and 17	NA	3	NA	3	q	medial	7	23445416-23632061	2 BAC Ends
zKp021H04	NA	NA	3	3	3	q	medial	NA		
zK178D09	3 and 23	NA	3	3	3	q	distal	3	22856698-23012289	2 BAC Ends
zK197E21	3	NA	3	NA	3	q	distal	3	15633176-15839847	2 BAC Ends
zC246E12	NA	NA	3	NA	3	q	distal	NA		
zK061N16	NA	NA	3	3	3	q	distal	NA		
zC122F08	U	3	3	NA	3	q	subtelomeric	3	67599924-67817005	2 BAC Ends
zK006B11	U	NA	3	NA	3	q	subtelomeric	3	65818529-65995191	2 BAC Ends
zC201B09	U	3	3	3	3	q	subtelomeric	3	65722937-65914853	2 BAC Ends
zC253B18	11	3	3	3	3	q	subtelomeric	3	3944043-3957172	1 BAC End
zK005H01	3	NA	NA	NA	3	q	subtelomeric	3	1010048-1023155	1 BAC End
zC274J07	15	3	3	3	3	q	subtelomeric	NA		
zC012P12	6	6	3	NA	3	q	telomeric	3	3345534-3358766	1 BAC End
zK030G05	3	NA	3	3	3	q	telomeric	3	1237564-1470750	2 BAC Ends
zC262N01	4	4	NA	4	4	p	telomeric	4	46297923-46311356	1 BAC End
zK030C13	18	NA	NA	NA	4	p	telomeric	18	41489856-41502975	1 BAC End
zC132P20	4	4	4	4	4	p	subtelomeric	4	1265901-1445384	2 BAC Ends
zC240J22	4	4 and 5	4	4	4	p	subtelomeric	4	4737249-4909691	2 BAC Ends
zK014D08	4	NA	4	4	4	p	subtelomeric	4	4998249-5262388	2 BAC Ends
zK201E17	4	NA	4	NA	4	p	subtelomeric	4	5766241-5779510	1 BAC End
zC276H18	4	4	4	NA	4	p	subtelomeric	4	8905645-8919023	1 BAC End
zK153K10	4	NA	4	4	4	p	subtelomeric	4	12283944-12466480	2 BAC Ends
zK061F09	4	NA	4	24	4	p	subtelomeric	NA		
zK021H14	4	NA	4	4	4	p	distal	4	8419099-8638977	2 BAC Ends
zK222F08	4	NA	4	4	4	p	distal	4	9301552-9509460	2 BAC Ends

zC239E06	NA	NA	4	4	4	p	distal	NA		
zK244B23	4	NA	4	4	4	p	medial	4	13375433-13571220	2 BAC Ends
zK180P18	4	NA	4	4	4	p	medial	4	16351073-16572320	2 BAC Ends
zC132J14	4	4	NA	NA	4	p	proximal	4	14480200-14480908	T51 RH Map
zK097C07	4	NA	4	4	4	p	proximal	4	32310830-32324182	1 BAC End
zC092L17	4	4	4	4	4	p	near the centromere	4	17086785-17100038	1 BAC End
zC260P03	4	4	4	4	4	p	near the centromere	4	18942123-19123022	2 BAC Ends
zK266P18	4	NA	4	NA	4	p	near the centromere	4	20774444-20787667	1 BAC End
zK066D18	4	NA	4	4	4	p	near the centromere	4	33728956-33902772	2 BAC Ends
zK210E13	4	NA	4	NA	4	p	near the centromere	4	33993532-34215037	2 BAC Ends
zC242E19	NA	14	NA	U	4	p	near the centromere	NA		
zC149P05	4	4	4	4	4	q	near the centromere	4	32064870-32078129	1 BAC End
zK009A20	4	NA	4	4	4	q	near the centromere	4	33006299-33230093	2 BAC Ends
zC091G03	4	4	4	4	4	q	near the centromere	4	33583684-33758324	2 BAC Ends
zK043F09	4	NA	NA	4	4	q	heterochromatic region	4	35578924-35735588	2 BAC Ends
zC128F02	15	4	NA	NA	4	q	heterochromatic region	4	37627935-37795193	2 BAC Ends
zC199M09	14	4, 5, 13, and 16	14	14	4	q	heterochromatic region	4	41492210-41505320	1 BAC End
zC207E19	4	3 and 4	4	4	4	q	heterochromatic region	4	41837915-42054373	2 BAC Ends
zK237G15	5, 8, and 10	NA	4	4	4	q	heterochromatic region	4	45630303-45643723	1 BAC End
zC039D08	3 and U	3	NA	U	4	q	heterochromatic region	3 and U	chr 3: 70199481-70212777	1 BAC End
									chr U: 15585436-15598732 15735130-15748438	1 BAC End
zK014O06	U	NA	3	3	4	q	heterochromatic region	3	70292200-70526076	2 BAC Ends
zK026M03	5 and 23	NA	NA	5	4	q	heterochromatic region	5	43808864-43963922	2 BAC Ends
zK169L05	U	NA	4	NA	4	q	heterochromatic region	6 and 21	chr 6: 9203223-9316482	2 BAC Ends
									chr 21: 49428636-4942923	T51 RH Map
zK020L04	4	NA	12	NA	4	q	heterochromatic region	12	1258781-1488424	2 BAC Ends
zC197F20	14	14	14	14	4	q	heterochromatic region	14	38145844-38344621	2 BAC Ends
zC218H08	U	14	14	14	4	q	heterochromatic region	14	42575952-42589232 55305423-55318503	1 BAC End
zK285E18	14	NA	14	14	4	q	heterochromatic region	14	42784590-42947367	2 BAC Ends

zK078O07	14	NA	14	14	4	q	heterochromatic region	14	52356148-52621658	2 BAC Ends
zC187D01	14	14	14	14	4	q	heterochromatic region	14	56995151-57171235	2 BAC Ends
zC196H24	14	14	14	14	4	q	heterochromatic region	14	59512626-59721463	2 BAC Ends
zK003P04	U	NA	14	14	4	q	heterochromatic region	14	69585234-69598345	1 BAC End
zC167J09	15	15	15	NA	4	q	heterochromatic region	15	32398231-32538362	2 BAC Ends
zK011L18	17	NA	17	17	4	q	heterochromatic region	17	16190913-16407002	2 BAC Ends
zC208F21	19	11, 18, and 19	14	14	4	q	heterochromatic region	19	42416256-42429550	1 BAC End
zK165E24	U	NA	NA	U	4	q	heterochromatic region	U	17196851-17210208<br>17366292-17379641	1 BAC End
zK238O14	U	NA	NA	U	4	q	heterochromatic region	U	674456-930935	2 BAC Ends
bZ009C18	NA	NA	NA	14	4	q	heterochromatic region	NA		
zC271G18	NA	7, 9, and 12	NA	U	4	q	heterochromatic region	NA		
zK082I20	NA	NA	NA	U	4	q	heterochromatic region	NA		
zC079A18	15	4	NA	NA	4	q	telomeric	4	37566365-37720847	2 BAC Ends
zC087E10	5	5	5	5	5	p	telomeric	5	75518198-75518629	T51 RH Map
zK002E19	5	NA	NA	NA	5	p	subtelomeric	5	67688527-67928972	2 BAC Ends
zK020J10	5	NA	5	5	5	p	distal	NA		
zK208B17	5	NA	5	NA	5	p	distal	NA		
zK007J10	U	NA	5	NA	5	p	medial	5	77840283-77853421	1 BAC End
zK007B18	5	NA	NA	NA	5	q	near the centromere	5	46977693-47128275	2 BAC Ends
zC077G09	5	5	5	NA	5	q	proximal	5	42403789-42551945	2 BAC Ends
zC132M05	5	5	NA	NA	5	q	medial	5	4138088-4309756	2 BAC Ends
zK125I20	5	NA	5	NA	5	q	medial	5	32281929-32456100	2 BAC Ends
zC117M20	5	5	5	17	5	q	medial	5	28392741-28571146	2 BAC Ends
zC114C12	5	5	5	5	5	q	medial	5	16102499-16260537	2 BAC Ends
zK035I22	5	NA	5	5	5	q	medial	NA		
zC001E03	5	5	NA	NA	5	q	distal	5	41906019-42082255	2 BAC Ends
zK061N21	U	NA	5	NA	5	q	distal	22 and U	chr 22: 30049270-30062364<br>chr U: 48876881-48889971	1 BAC End<br>1 BAC End
zK210G19	5	NA	5	5	5	q	subtelomeric	5	50872193-51123587	2 BAC Ends
zC207K07	NA	NA	NA	NA	5	q	subtelomeric	NA		
zC150K20	8	5 and 7	5	NA	5	q	telomeric	5	1507291-1520533	1 BAC End

zK023D07	6	NA	6	6	6	p	medial	6	11471266-11687049	2 BAC Ends
zC127M10	6	6	6	6	6	p	proximal	6	4251867-4438662	2 BAC Ends
zC186M12	16	NA	6	6	6	p	proximal	NA		
zK011H02	6	NA	6	6	6	p	proximal	NA		
zC210N21	6	6	NA	NA	6	p	near the centromere	15	57312283-57474348	2 BAC Ends
zC122J16	14	14, 17, and 25	14	1	6	q	near the centromere	14	55842747-56015589	2 BAC Ends
zC002A23	U	U	NA	U	6	q	near the centromere	U	13999754-14170910	2 BAC Ends
zC207G04	NA	NA	NA	U	6	q	near the centromere	NA		
zK210O07	25	NA	6	6	6	q	proximal	6	49699243-49712539	1 BAC End
zK038M05	6	NA	6	6	6	q	proximal	NA		
zC223I03	6	6	6	NA	6	q	medial	NA		
zK148L02	17	NA	6	6	6	q	distal	6	37986472-38178432	2 BAC Ends
zC060H08	U	6	NA	NA	6	q	subtelomeric	6	64385164-64547964	2 BAC Ends
zK166J19	16	NA	6	NA	6	q	telomeric	16	48632997-48646230	1 BAC End
zK009M06	7	NA	NA	NA	7	p	telomeric	7	81423473-81424057	T51 RH Map
zC139O04	NA	13	NA	U	7	p	subtelomeric	NA		
zC251B22	NA	7	7	7	7	p	subtelomeric	NA		
zK046I09	7	NA	7	NA	7	p	medial	7	83011172-83235010	2 BAC Ends
zC219K13	7	7	7	NA	7	p	near the centromere	NA		
zC069P21	7	3 and 7	7	7	7	q	near the centromere	7	58557812-58721634	2 BAC Ends
zK014N10	7	NA	NA	NA	7	q	near the centromere	7	53070009-53309139	2 BAC Ends
zC113K19	NA	NA	7	7	7	q	near the centromere	NA		
zK090B24	7	NA	7	NA	7	q	near the centromere	NA		
zC150J10	7	7 and 11	7	7	7	q	medial	7 and 11	chr 7: 36458590-36471865	1 BAC End
									chr 11: 21346088-21359066	1 BAC End
zC251J08	7	7	7	7	7	q	medial	7	36832720-36845803	1 BAC End
zK249N13	7	NA	7	NA	7	q	medial	7	36978381-36991760	1 BAC End
zK265M08	7	NA	7	NA	7	q	subtelomeric	7	15384731-15554024	2 BAC Ends
zC209N17	NA	NA	NA	U	7	q	telomeric	NA		
zC128L16	7	7	NA	NA	7	q	telomeric	7	1387156-1400357	1 BAC End
zC212I24	7	7	7	7	7	q	telomeric	7	455951-469215	1 BAC End
zC261I17	U	U	NA	U	7	q	telomeric	U	7933272-8107445	2 BAC Ends
zK016N15	8	NA	8	8	8	p	telomeric	8	64875251-65092668	2 BAC Ends
zC057O07	8	8	8	NA	8	p	telomeric	8	64775008-64949315	2 BAC Ends
zC069A12	8	8	8	NA	8	p	telomeric	8	56536959-56837752	2 BAC Ends
zK220O05	8 and 20	NA	NA	8	8	p	telomeric	8 and 20	chr 8: 51932153-51945630	1 BAC End
									chr 20: 43012365-43025703	1 BAC End
zC080A15	8	8	8	8	8	p	distal	8	15923971-15937163	1 BAC End
zK067I21	8	NA	8	NA	8	p	near the centromere	8	48979468-48992522	1 BAC End
zC255F14	NA	NA	8	8	8	p	near the centromere	NA		

zC103G04	8	7 and 8	NA	NA	8	q	near the centromere	8	27341164-27354413<br>27754804-27768077	1 BAC End
zC106G04	8	8	8	8	8	q	proximal	8	31551090-31742063	2 BAC Ends
zC163L21	8	8	8	8	8	q	proximal	8	26998281-27011372	1 BAC End
zK283N20	20	NA	20	20	8	q	medial	20	39503626-39648349	2 BAC Ends
zK014L10	8	NA	8	NA	8	q	medial	10	21964251-22306546	2 BAC Ends
zK188E21	NA	NA	20	20	8	q	medial	NA		
zC269M15	8	8	8	8	8	q	distal	8	27677838-27818484	2 BAC Ends
zC027L17	8	8 and 18	NA	NA	8	q	subtelomeric	8	13333796-13347073	1 BAC End
zK149F22	8	NA	8	8	8	q	telomeric	8 and 10	chr 8: 56961312-56974658	1 BAC End
									chr 10: 16685649-16698995	1 BAC End
zK049H09	8	NA	8	8	8	q	telomeric	8	828816-1040252	2 BAC Ends
zC115B08	9	9	9	NA	9	p	telomeric	9	3840695-3853990<br>4229898-4243637	1 BAC End
zC166E11	13, 19, and 20	19 and 20	9	NA	9	p	telomeric	13, 19, and 20	chr 13: 32063634-32076929	1 BAC End
									chr 19: 34016152-34029427	1 BAC End
									chr 20: 61597942-61611225	1 BAC End
zK249G23	NA	NA	9	9	9	p	distal	9	3211227-3224688	1 BAC End
zC067P04	NA	9	9	NA	9	p	distal	9	7024085-7202223	2 BAC Ends
zC121J05	9	9	9	9	9	p	distal	9	7029022-7042325	1 BAC End
zK120M04	9	NA	9	9	9	p	proximal	9	7572965-7781353	2 BAC Ends
zK001A09	9	NA	NA	NA	9	q	near the centromere	9	12737861-12986999	2 BAC Ends
zC212N06	9 and 11	9	9	9	9	q	near the centromere	9	16985534-17290962	2 BAC Ends
zC173O04	1 and 17	9	9	NA	9	q	proximal	9	15765894-15947715	2 BAC Ends
zK179E14	7	NA	9	NA	9	q	proximal	NA		
zC004P07	9	9	NA	NA	9	q	medial	9	31120151-31133418	1 BAC End
zK025L23	9	NA	9	9	9	q	medial	9	35078811-35314831<br>35079624-35314831	2 BAC Ends
zC085P24	9	9	9	NA	9	q	distal	9	25869080-25882314	1 BAC End
zC165B19	9	9	9	9	9	q	distal	9	29705084-30050905<br>29876195-30050905	2 BAC Ends
zK255G14	NA	NA	9	NA	9	q	distal	NA		
zC012N08	11	9	19	9	9	q	telomeric	9	51550658-51563901<br>51736420-51749663	1 BAC End
zC066E19	U	14	9	9	9	q	telomeric	U	2561846-2724314	2 BAC Ends
zC133D02	5 and 23	10	10	10	10	p	telomeric	10	269080-432849	2 BAC Ends
zC128P08	10	10	NA	NA	10	p	telomeric	10	272675-273165	T51 RH Map
zC242F23	15	1 and 10	10	NA	10	p	telomeric	1 and 10	chr 10: 2516177-2529347	1 BAC End
									chr 1: 66508224-66521481	1 BAC End
zK024N17	U	NA	10	10	10	p	telomeric	10	4774567-4787656	1 BAC End

zC093H16	U	9 and 10	10	10	10	p	telomeric	10	4935623-4948940	1 BAC End
zK171L20	9 and 10	NA	10	NA	10	p	telomeric	10	48893995-49106495	2 BAC Ends
zK224O08	NA	NA	10	NA	10	p	telomeric	NA		
zC197A16	10	10	10	NA	10	p	medial	10	9207989-9404988	2 BAC Ends
zK030A08	10	NA	NA	NA	10	p	medial	10	10123547-10332196	2 BAC Ends
zC043O18	8	14	NA	NA	10	p	proximal	14	7200721-7214143	1 BAC End
zK047C08	10 and 23	NA	23	NA	10	p	proximal	23	19470951-19484524	1 BAC End
zC136O04	10	10	10	10	10	q	near the centromere	10	17320034-17333302	1 BAC End
zK003B23	10	NA	10	NA	10	q	near the centromere	NA		
zC149N01	10	10	10	NA	10	q	medial	10	21969495-21982757	1 BAC End
zK056F11	10	NA	10	NA	10	q	medial	10	26675763-26689052	1 BAC End
zK161J10	NA	NA	10	10	10	q	medial	NA		
zC135J07	10	10	10	10	10	q	distal	10	41455896-41640209	2 BAC Ends
zK016F21	5	NA	10	10	10	q	subtelomeric	10	38651220-38664339<br>39166105-39179295	1 BAC End
zC022E09	10	10	10	NA	10	q	telomeric	10	45339834-45353078	1 BAC End
zC159E12	1 and 22	11	11	11	11	p	telomeric	NA		
zK113M09	11	NA	11	NA	11	p	subtelomeric	11	3967064-3982579<br>6269256-6282578	1 BAC End
zC108O08	11	11	NA	NA	11	p	subtelomeric	11	4304693-4492412	2 BAC Ends
zC270L17	NA	11	NA	11	11	p	subtelomeric	NA		
zK008J21	11	NA	NA	NA	11	p	near the centromere	11, 14, and 15	chr 11: 7710723-7713378	T51 RH Map
									chr 14: 7655889-7669030	1 BAC End
									chr 15: 21033550-21046667<br>21348107-21361224	1 BAC End
zK015K08	11	NA	NA	NA	11	p	near the centromere	11	12463167-12476269<br>13084746-13097893	1 BAC End
zK014H17	11	NA	11	NA	11	q	near the centromere	2 and 11	chr 11: 17450218-17463410	1 BAC End
									chr 2: 60976060-60989206	1 BAC End
zC042L14	11	6 and 11	NA	NA	11	q	near the centromere	6 and 11	chr 11: 19858955-19872247	1 BAC End
									chr 6: 16151829-16165096	1 BAC End
zC086H15	11	11	11	11	11	q	near the centromere	11	21613791-21627048	1 BAC End
zC118E09	11	11	11	NA	11	q	near the centromere	11	23309557-23322825<br>23784252-23797513	1 BAC End
zK029P20	11	NA	11	NA	11	q	proximal	11	25981060-25994193<br>26637522-26650665	1 BAC End
zC267N08	NA	U	NA	NA	11	q	proximal	NA		
zK029F02	11	NA	11	11	11	q	proximal	NA		
zK189A09	11	NA	11	11	11	q	medial	11	30699723-30712930	1 BAC End
zC107E16	11	11	11	11	11	q	medial	11	32419412-32574159	2 BAC Ends

zK111H12	11	NA	11	NA	11	q	medial	NA			
zK013A21	11	NA	11	NA	11	q	distal	11	26116159-26129275	1 BAC End
zC171E12	11	11	11	NA	11	q	subtelomeric	11	42855790-42996502	2 BAC Ends
zC153M01	11	11	NA	11	11	q	subtelomeric	11	6499169-6667126	2 BAC Ends
									6501295-6667126	
zK202E22	NA	NA	11	11	11	q	subtelomeric	NA		
zC115I06	11	11	NA	NA	11	q	telomeric	11	46876389-47031952	2 BAC Ends
									46876389-47034560	
zC266K22	14	14	11	11	11	q	telomeric	14	76763889-76777255	1 BAC End
zK208K08	NA	NA	NA	NA	11	q	telomeric	NA		
zC121C04	12	12	NA	NA	12	p	telomeric	U	129605838-129619147	1 BAC End
zC093E11	12	12	12	12	12	p	medial	12	4713843-4880901	2 BAC Ends
zC093M08	12	12	NA	U	12	p	medial	12	12725634-12738935	1 BAC End
zK025P11	20	NA	12	12	12	p	medial	NA		
zK014I09	20	NA	20	20	12	p	proximal	20	9773096-9964928	2 BAC Ends
zK016I05	12	NA	12	12	12	p	proximal	12	18787218-19011844	2 BAC Ends
zC176J23	U	12	12	NA	12	p	near the centromere	12	55862742-55876003	1 BAC End
zK021M21	12	NA	12	NA	12	q	near the centromere	12	24430420-24443538	1 BAC End
									25667949-25681070	
zK190E10	12	NA	14	U	12	q	near the centromere	12	31768612-32024946	2 BAC Ends
									31859633-32024946	
zK022H21	12	NA	NA	NA	12	q	near the centromere	NA		
zC225J11	12	12	12	NA	12	q	proximal	12	15378316-15481280	2 BAC Ends
zC244C18	12	12	12	12	12	q	proximal	NA		
zC192N18	12	12	12	12	12	q	medial	12	23362809-23529950	2 BAC Ends
zC079A21	12	12	12	NA	12	q	distal	12	29703056-29716209	1 BAC End
zC160A03	12	12	12	12	12	q	distal	12	44734569-44905782	2 BAC Ends
zC086E02	3	12	NA	NA	12	q	telomeric	12	49665073-49825676	2 BAC Ends
zK006L12	13	NA	NA	NA	13	p	telomeric	13 and 18	chr 13: 473169-486297	1 BAC End
									chr 18: 55768933-55782015	1 BAC End
zC250O01	13	13	13	13	13	p	telomeric	13	793689-806954	1 BAC End
zC101N13	13	13	13	13	13	p	telomeric	13	29124961-29298009	2 BAC Ends
zC243F03	NA	NA	13	13	13	p	distal	NA		
zK022N17	13	NA	13	NA	13	p	medial	13	22474499-22809338	2 BAC Ends
zK038M12	13	NA	13	NA	13	p	proximal	13	29127200-29140342	1 BAC End
zK158P02	NA	NA	13	13	13	p	proximal	NA		
zK016I06	13	NA	13	NA	13	p	near the centromere	13	23463134-23672201	2 BAC Ends
zC206K20	13	13	2	13	13	q	proximal	13	38973361-39322543	2 BAC Ends
									39166131-39322543	
zK011L06	13	NA	NA	NA	13	q	proximal	13	41535602-41768155	2 BAC Ends

zC202M24	13	13	13	NA	13	q	medial	13	37670918-37684238	1 BAC End
zK162B03	NA	NA	13	13	13	q	medial	NA		
zK015F17	NA	NA	13	13	13	q	distal	NA		
zC232K09	NA	13	13	NA	13	q	subtelomeric	13	18180736-18351259	2 BAC Ends
zK167E04	13	NA	13	NA	13	q	subtelomeric	13	44928834-45129012	2 BAC Ends
zK018N13	13	NA	13	NA	13	q	subtelomeric	13	51691753-51704871	1 BAC End
zC229G14	19	11	9	9	13	q	subtelomeric	11	43632779-43645893	1 BAC End
zC065J24	19	13	NA	NA	13	q	telomeric	13	50118106-50118856	T51 RH Map
zC024F03	2 and 13	2 and 13	NA	NA	13	q	telomeric	2 and 13	chr 13: 56288803-56302059<br>chr 2: 47652278-47665540	1 BAC End<br>1 BAC End
zC134I04	13	13	NA	NA	13	q	telomeric	13	58903541-59210716	2 BAC Ends
zC117H14	13	13	NA	NA	13	q	telomeric	13	59455129-59468420<br>59808623-59821942	1 BAC End
zC030I02	13	13	13	13	13	q	telomeric	13	59467592-59480806<br>59468732-59481945	1 BAC End
zC258M18	1	1	NA	NA	13	q	telomeric	1	12694677-12708340<br>13056013-13069537	1 BAC End
zC189P15	1 and 6	1 and 6	NA	NA	13	q	telomeric	1 and 6	chr 1: 18705093-18718377<br>chr 6: 6806311-6819595	1 BAC End<br>1 BAC End
zC234K01	2	2 and 6	NA	NA	13	q	telomeric	2	47652266-47665540	1 BAC End
zK018P03	2 and 6	NA	NA	NA	13	q	telomeric	6	6808446-6821597	1 BAC End
zC344P05	NA	NA	NA	NA	13	q	telomeric	NA		
zC113I09	NA	NA	11	11	13	q	telomeric	NA		
zC117N19	14	14	NA	NA	14	p	telomeric	14	2011198-2024532<br>2713791-2727076	1 BAC End
zC198I06	NA	14	5	NA	14	p	telomeric	NA		
zC207O21	14	14	14	14	14	p	distal	14	3088523-3323703	2 BAC Ends
zC246K16	NA	NA	14	NA	14	p	distal	NA		
zC261F09	U	U	NA	U	14	p	medial	U	7368479-7382147	1 BAC End
zC194M19	14	14	14	NA	14	p	medial	NA		
zK169H01	14 and 22	NA	14	14	14	p	proximal	14	15756834-15929925	2 BAC Ends
zC159I08	14	14	14	14	14	p	proximal	14	19142113-19155387	1 BAC End
zK170I11	14	NA	14	14	14	p	near the centromere	14	7439763-7622786	2 BAC Ends
zC117E17	8 and 14	14	NA	NA	14	p	near the centromere	14	29784131-29965843	T51 RH Map
zC117I07	8 and 14	14	14	NA	14	q	near the centromere	NA		
zC125N22	14	14	NA	NA	14	q	telomeric	14	84157968-84158959	T51 RH Map
zK077E06	15	NA	NA	NA	15	p	telomeric	15	4319660-4520645	2 BAC Ends
zC055C01	15	15	NA	NA	15	p	telomeric	15	6586947-6600244<br>7319875-7333164	1 BAC End
zC125H09	15	15	NA	NA	15	p	medial	15	21472800-21651124	2 BAC Ends

zK202L22	3 and 13	NA	NA	NA	15	p	near the centromere	3 and 13	chr 3: 16103272-16116652<br>chr 13: 54577880-54591216	1 BAC End<br>1 BAC End
zC132F07	15	15	15	15	15	q	near the centromere	15	15628811-15807466	2 BAC Ends
zC024O10	15	15	15	15	15	q	near the centromere	NA		
zC125M22	NA	6	6	6	15	q	near the centromere	NA		
zC120C08	15	15	15	15	15	q	near the centromere	15	27510491-27766996	2 BAC Ends
zK001O01	15	NA	15	15	15	q	proximal	15	1337915-1562891	2 BAC Ends
zK257C10	15	NA	15	15	15	q	proximal	15	31545805-31804299	2 BAC Ends
zC191A16	15	15	15	15	15	q	subtelomeric	15	51550363-51698204	2 BAC Ends
zK151P21	15	NA	15	15	15	q	telomeric	15	33936194-34257286	2 BAC Ends
zK042L23	15	NA	15	NA	15	q	telomeric	15	50261409-50274503	1 BAC End
zC059M05	15	15	NA	NA	15	q	telomeric	15	50588681-50786799	2 BAC Ends
zK019A16	22	NA	22	22	15	q	telomeric	22	6439750-6660019	2 BAC Ends
zC119D19	5	16	16	16	16	p	telomeric	16	9328662-9341914	1 BAC End
zC127P21	13	13	NA	NA	16	p	telomeric	13	36482408-36641865	2 BAC Ends
zK011F05	16	NA	16	16	16	p	subtelomeric	16	7569612-7846417	2 BAC Ends
zK014I07	16	NA	NA	NA	16	p	distal	16	11895536-11908673	1 BAC End
zC106K04	16	16	16	NA	16	p	medial	16	11757114-11964371	2 BAC Ends
zK082O10	9	NA	16	NA	16	p	medial	11	16247354-16260641	1 BAC End
zK053P21	16	NA	16	16	16	p	proximal	16	28737066-28750400	1 BAC End
zC175G06	16	16	16	16	16	p	proximal	16	36396657-36538512	2 BAC Ends
zC132M17	16	16	NA	NA	16	q	near the centromere	16	28079663-28343689	2 BAC Ends
zC213B07	16	16	16	16	16	q	near the centromere	16	34049577-34062864	1 BAC End
zC167H02	16	16	16	16	16	q	near the centromere	16	3676766-3814241	2 BAC Ends
zC105C13	16	16	16	16	16	q	proximal	NA		
zC236O11	16	16	16	NA	16	q	medial	16	42059420-42072702<br>44390350-49036330<br>44519560-445328323	1 BAC End
zK201I06	16	NA	16	16	16	q	medial	16	49023040-49036330	1 BAC End
zH070I04	NA	NA	16	NA	16	q	medial	NA		
zK034D22	NA	NA	16	16	16	q	medial	NA		
zK242E21	16	NA	16	16	16	q	distal	16	49982457-50144220	2 BAC Ends
zK107O06	16	NA	16	16	16	q	distal	16	57421371-57434707	1 BAC End
zK255N06	16	NA	16	16	16	q	subtelomeric	16	47456229-47692912	2 BAC Ends
zK014E01	U	NA	NA	U	16	q	subtelomeric	U	16301578-16314719	1 BAC End
zK246M23	15	NA	16	NA	16	q	telomeric	16	56833579-57022084	2 BAC Ends
zC121P03	9	11	NA	NA	16	q	telomeric	16	61408707-61409075	T51 RH Map
zK013L17	17	NA	NA	NA	17	p	telomeric	17	61034178-61047182	1 BAC End
zC018F08	U	17	NA	NA	17	p	medial	17	58730073-58812259	2 BAC Ends
zK006P15	17	NA	NA	NA	17	q	near the centromere	17	42580390-42744764	T51 RH Map

zC195B21	NA	NA	17	17	17	q	near the centromere	NA		
zK014B13	17	NA	NA	NA	17	q	proximal	17	324810-337877	1 BAC End
zK170L10	NA	NA	17	17	17	q	proximal	NA		
zC194D01	17	17	17	17	17	q	medial	17	53014210-53212372	2 BAC Ends
zC210P22	17 and 21	17	17	17	17	q	medial	17	44610301-44756567	2 BAC Ends
zC160F23	17	17	17	17	17	q	medial	17	38476103-38638750	2 BAC Ends
zC077G03	19	17	NA	NA	17	q	medial	17	15321079-15321770	T51 RH Map
zC133L14	17	17	17	17	17	q	distal	17	18039549-18202654	2 BAC Ends
zC074A06	17	17	NA	NA	17	q	distal	17	1526712-1678416<br>48172473-48185770	2 BAC Ends<br>T51 RH Map
zK038J10	17	NA	17	17	17	q	distal	NA		
zC077G19	17	17	17	17	17	q	subtelomeric	17	6482566-6669339	2 BAC Ends
zC042B22	U	17	17	17	17	q	telomeric	17	5393544-5573088<br>5394563-5573088	2 BAC Ends
zC241L24	18	18	18	NA	18	p	telomeric	18	49605027-49761299<br>49605027-49794159	2 BAC Ends
zC095I06	18	18	NA	NA	18	p	telomeric	18	46125632-46279646	2 BAC Ends
zK079N12	NA	NA	18	NA	18	p	telomeric	NA		
zK003B08	18	NA	18	18	18	p	distal	18	38606591-38619691<br>55577034-55590134	1 BAC End
zC255L12	NA	NA	18	18	18	p	distal	NA		
zK005J13	18	NA	NA	NA	18	p	near the centromere	18	39825600-40035877	2 BAC Ends
zK150H10	18	NA	18	U	18	p	near the centromere	18	39751050-39764387	1 BAC End
zK180N16	19	NA	18	U	18	p	near the centromere	18	35810109-35823451	1 BAC End
zC122K23	18	18	18	18	18	q	near the centromere	18	28798942-28971494	2 BAC Ends
zK016F19	18	NA	18	NA	18	q	proximal	18	23810982-24018828	2 BAC Ends
zK207D05	18	NA	18	NA	18	q	proximal	18	18369631-18382992	1 BAC End
zH124C24	18	NA	18	18	18	q	proximal	NA		
zK286O23	18	NA	18	NA	18	q	distal	18	14889191-14902514	1 BAC End
zK263N17	18	NA	18	NA	18	q	distal	18	13264338-13563907	2 BAC Ends
zC234N07	18 and 23	18	18	18	18	q	distal	NA		
zK283N16	18	NA	18	18	18	q	distal	NA		
zK180H12	18	NA	18	18	18	q	telomeric	18	9745594-9758933	1 BAC End
zK014D24	18	NA	NA	NA	18	q	telomeric	18	4273066-4286213	1 BAC End
zK089B17	19	NA	19	19	19	p	telomeric	19	5083126-5096357	1 BAC End
zC039E15	19	1, 15, 17, and 19	19	19	19	p	telomeric	19	5999779-6170827	2 BAC Ends
zK263H23	20	NA	19	19	19	p	distal	19	1055716-1068859	1 BAC End
zK218F09	18	NA	19	19	19	p	distal	19	4435068-4598775	2 BAC Ends
zKp024A07	NA	NA	19	19	19	p	distal	NA		
zC234F14	NA	NA	19	19	19	p	medial	NA		

zC245H07	NA	NA	19	19	19	p	medial	NA		
zK222B08	19	NA	19	19	19	p	proximal	2	11924256-11937604	1 BAC End
zC001J13	19	19	19	19	19	p	proximal	19	15199786-15378202	2 BAC Ends
zK219L12	19	NA	19	19	19	p	proximal	19	23727698-23741070	1 BAC End
zC143E20	19	19	19	NA	19	p	proximal	19	32158503-32171765	1 BAC End
zC132A16	19	19	NA	NA	19	p	near the centromere	19	37098897-37446879	2 BAC Ends
zK025E11	19	NA	19	19	19	q	near the centromere	19	13834242-14040211	2 BAC Ends
zC239D06	14	19	19	19	19	q	near the centromere	19	19919723-19932793	1 BAC End
zC195B13	12	12	19	19	19	q	near the centromere	12	23223873-23385646	2 BAC Ends
zC254E15	19	19	19	19	19	q	medial	19	44513909-44653658	2 BAC Ends
bZ045K05	19	NA	19	19	19	q	medial	NA		
zC194E15	19	19	19	19	19	q	subtelomeric	19	31956265-32171765	2 BAC Ends
zK201G07	21	NA	19	NA	19	q	telomeric	19	42699599-42712972	1 BAC End
zC036I10	19	19	NA	NA	19	q	telomeric	19	49766723-49932919	2 BAC Ends
zC118G14	20	20	NA	NA	20	p	telomeric	20	426186-439563	T51 RH Map
zK077B17	NA	NA	20	20	20	p	telomeric	NA		
zC236C21	NA	20	20	NA	20	p	subtelomeric	NA		
zC257G08	NA	NA	20	20	20	p	subtelomeric	NA		
zC241J12	20	20	20	20	20	p	distal	20	203076-361689	2 BAC Ends
zK060A16	20	NA	20	20	20	p	distal	NA		
zKp059G05	NA	NA	20	NA	20	p	distal	NA		
zC272F15	20	20	20	20	20	p	medial	20	3895697-3909120<br>4067776-4081199	1 BAC End
zK242M13	5	NA	NA	5	20	p	medial	5	47857283-47870671	1 BAC End
zK080I09	2	NA	NA	NA	20	p	medial	NA		
zK023K06	20	NA	20	20	20	p	proximal	20	28626656-28873284	2 BAC Ends
zC045M15	20	20	20	NA	20	p	proximal	20	3396516-3704807<br>3396516-3701195	2 BAC Ends
zC191D07	20	20	20	20	20	p	near the centromere	NA		
zK019F21	NA	NA	20	20	20	p	near the centromere	NA		
zK038F05	18	NA	NA	NA	20	q	near the centromere	18	19289634-19302754	1 BAC End
zK033I22	20	NA	20	20	20	q	near the centromere	20	20283093-20520186	2 BAC Ends
zK015B08	20	NA	NA	NA	20	q	near the centromere	20	24200016-24449760	2 BAC Ends
zK181I03	20	NA	20	20	20	q	proximal	20	1423595-1436881	1 BAC End
zC214H24	1 and 20	20	20	NA	20	q	proximal	20	22047422-22290255	2 BAC Ends
zK219E20	20	NA	20	20	20	q	proximal	20	24580213-24740749	2 BAC Ends
zK014A07	20	NA	20	20	20	q	medial	20	51816709-51829794	1 BAC End
zC195D17	3 and 20	16	20	20	20	q	medial	16	27366713-27469831<br>27366713-27576225	2 BAC Ends
zC266I18	20	20	20	NA	20	q	medial	NA		
zK025E12	20	NA	20	20	20	q	distal	20	31724992-31935130	2 BAC Ends

zK097O05	20	NA	20	20	20	q	distal	20	35201568-35214932	1 BAC End
zC063O20	20	20	20	20	20	q	distal	20	36091784-36256857	2 BAC Ends
zK221H15	20	NA	20	20	20	q	distal	20	46050421-46237734	2 BAC Ends
zC180B22	20	11 and 20	20	20	20	q	distal	20	57062587-57213698	2 BAC Ends
zK241L07	20	NA	20	20	20	q	distal	20	57737765-57920906	2 BAC Ends
zC153J24	20	20	20	20	20	q	telomeric	20	55135058-55316460	2 BAC Ends
zC134L13	20	20	NA	NA	20	q	telomeric	20	57235006-57235350	T51 RH Map
zC122A16	21	21	NA	NA	21	p	telomeric	21	48612405-48773329	2 BAC Ends
zK235H13	NA	NA	NA	U	21	p	telomeric	NA		
zK287G19	21	NA	21	NA	21	p	subtelomeric	21	49247171-49429313	2 BAC Ends
zC065O02	21	21	NA	NA	21	p	distal	21	46082212-46082906	T51 RH Map
zK036I03	21	NA	21	NA	21	p	distal	19 and 21	chr 21: 44937821-44950936	1 BAC End
									chr 19: 43010078-43023207	1 BAC End
zK192J13	NA	NA	21	NA	21	p	near the centromere	NA		
zC051A20	21	21	21	21	21	q	near the centromere	21	19908772-20113260	2 BAC Ends
zK125B07	NA	NA	21	21	21	q	medial	NA		
zK202I08	U	NA	21	NA	21	q	distal	21	9468945-9482256	1 BAC End
zK190E02	21	NA	21	NA	21	q	distal	NA		
zK014M09	21	NA	NA	NA	21	q	telomeric	21	128465-141586	1 BAC End
zK002J07	22	NA	NA	NA	22	p	telomeric	22	45560686-45761213	2 BAC Ends
zC079M20	22	22	22	22	22	p	telomeric	22	38500984-38514273	1 BAC End
									39246105-39259394	
zK250M06	9	NA	10	22	22	p	distal	4 and 22	chr 22: 46275546-46288888	1 BAC End
									chr 4: 32310036-32325665	1 BAC End
zK037M08	22	NA	22	22	22	p	distal	22	44689203-44860594	2 BAC Ends
zK169I05	22	NA	22	22	22	p	medial	22	40114710-40386583	2 BAC Ends
zC194K01	22	22	22	22	22	p	proximal	22	39643860-39829532	2 BAC Ends
zK232H18	NA	NA	22	22	22	p	proximal	NA		
zK004C23	22	NA	22	22	22	p	near the centromere	22	29343834-29356974	1 BAC End
zC132L16	22	22	NA	NA	22	q	near the centromere	22	29699587-29892716	2 BAC Ends
zC206A19	22	22	22	22	22	q	near the centromere	22	23282486-23445108	2 BAC Ends
zC197A02	22	22	22	22	22	q	near the centromere	22	21171187-21356597	2 BAC Ends
zC171D11	22	22	22	NA	22	q	near the centromere	22	16684025-16834881	2 BAC Ends
zC197G15	22	22	22	22	22	q	proximal	22	26670630-26877695	2 BAC Ends
zK222F02	17	NA	22	22	22	q	proximal	NA		
zK240E12	NA	NA	22	22	22	q	proximal	NA		
zK078L04	NA	NA	22	22	22	q	medial	NA		
zK192M14	22	NA	22	22	22	q	distal	22	16673790-16928514	2 BAC Ends
zC248M18	22	22	22	22	22	q	distal	22	16069511-16242025	2 BAC Ends
									16069511-16240801	
zK004C15	22	NA	22	22	22	q	distal	22	3234712-3247830	1 BAC End

zC246M06	NA	NA	22	22	22	q	distal	NA		
zC103F16	22	22	22	NA	22	q	subtelomeric	22	11277011-11435032	2 BAC Ends
zC168K08	22	22	22	22	22	q	subtelomeric	22	10898236-11022754	2 BAC Ends
zK020I20	22	NA	22	22	22	q	subtelomeric	22	3409145-3639844	2 BAC Ends
zKp079B07	22	22	22	22	22	q	subtelomeric	22	2931577-3122823	2 BAC Ends
zC009D01	19 and 22	10 and 22	NA	NA	22	q	telomeric	22	5382679-5395862<br>5774734-5787917	1 BAC End
zC118M01	17 and 22	22	22	22	22	q	telomeric	22	3558268-3716759	2 BAC Ends
zK015H08	NA	NA	22	22	22	q	telomeric	NA		
zC041B11	23	23	NA	NA	23	p	telomeric	23	5616496-5774855	2 BAC Ends
zC220I18	23	23	23	23	23	p	subtelomeric	23	9731503-9948563	2 BAC Ends
zC051C19	23	7 and 23	NA	NA	23	p	near the centromere	23	14938690-15102787	2 BAC Ends
zC195I16	U	7	23	23	23	q	near the centromere	7	39049299-39157681	2 BAC Ends
zC226M16	23	23	23	23	23	q	near the centromere	23	51849382-51862660	1 BAC End
zK151G10	23	NA	23	23	23	q	proximal	23	27629780-27788285	2 BAC Ends
zK166N08	23	NA	23	23	23	q	medial	23	31445054-31458401<br>31835829-31849176	1 BAC End
zC087H10	23	23	23	NA	23	q	medial	NA		
zC059K08	6	6, 8, 19, and 23	6	NA	23	q	telomeric	6	52349901-52363213	1 BAC End
zC214H13	NA	NA	23	23	23	q	telomeric	NA		
zK022E19	24	NA	NA	NA	24	p	telomeric	24	1829835-1842955	1 BAC End
zK018N12	24	NA	24	24	24	p	telomeric	24	4856363-5099040	2 BAC Ends
zC261O07	U	24	24	24	24	p	subtelomeric	U	5830296-5975505	2 BAC Ends
zK001A04	24	NA	NA	NA	24	p	medial	24	8573267-8884262	2 BAC Ends
zC014G13	NA	NA	24	24	24	q	near the centromere	NA		
zC161H07	NA	24	NA	24	24	q	near the centromere	NA		
zK001P06	24	NA	24	24	24	q	medial	24	26124047-26137165	1 BAC End
zC089L23	NA	3 and 7	24	NA	24	q	medial	NA		
zK162H01	NA	NA	24	24	24	q	medial	NA		
zK226L10	NA	NA	24	24	24	q	medial	NA		
zK124I03	24	NA	24	NA	24	q	telomeric	24	9909626-9922970	1 BAC End
zC118G02	17 and 24	24	NA	NA	24	q	telomeric	24	40764335-40921293<br>40764335-40955554	2 BAC Ends
zK021G19	NA	NA	NA	24	24	q	telomeric	NA		
zC096F02	25	25	NA	NA	25	p	telomeric	25	33175593-33340497	2 BAC Ends
zKp007F01	NA	NA	25	25	25	p	medial	NA		
zK019L19	U	NA	25	NA	25	p	near the centromere	25	26763122-26776263	1 BAC End
zK155H01	13	NA	25	NA	25	q	near the centromere	25	25812239-25825490	1 BAC End
zC220G18	25	25	25	25	25	q	near the centromere	25	24647669-24660986	1 BAC End
zK044K01	U	NA	25	25	25	q	near the centromere	25	13078916-13092063	1 BAC End

zC059O09	25	25	25	25	25	q	near the centromere	25	11514557-11527868<br>31179510-31192771	1 BAC End
zC226K23	25	25	25	25	25	q	proximal	25	10657370-10670645	1 BAC End
zC059G12	25	25	NA	NA	25	q	medial	25	17075400-17088685<br>17977447-17990723<br>18182542-18195818	1 BAC End
zK007N16	25	NA	25	25	25	q	subtelomeric	25	6124060-6137170	1 BAC End
zC087L10	25	25	NA	NA	25	q	telomeric	25	3400941-3401116	T51 RH Map

*Based upon data obtained as of August 2006

†Mapped unambiguously to an unknown chromosome (chromosome U)

‡NA: BAC clone is not in the database
