# Supplementary material for: Definition of the zebrafish genome using flow cytometry and cytogenetic mapping
Source: BMC Genomics. 2007 Jun 27;8:195. doi: 10.1186/1471-2164-8-195 (PMC1925092; doi:10.1186/1471-2164-8-195)
Supplement: Additional file 2 — A table summarizing the concordance of the chromosomal locations assigned by cytogenetic mapping with the most current build of the zebrafish genome assembly (Zv6-as of May 2007). [file 1471-2164-8-195-S2.pdf]

Additional file 2. A table summarizing the concordance of the chromosomal locations assigned by cytogenetic mapping with the most current build of the zebrafish genome assembly (Zv6-as of May 2007).

	No. of clones*	Percent of clones
Clones not in UCSC database	109	21.4
Clones present in UCSC database	401	78.6
Agree	332	82.8
Disagree	41	10.2
Map to multiple chromosomes by database and at least 1 agree	13	3.3
Map to multiple chromosomes by database and none agree	6	1.5
Mapped to "U" †	9	2.2

*Total number of clones based upon the 510 BAC clones assigned to a unique LG chromosome by FISH mapping

†Mapped unambiguously to an unknown chromosome (chromosome U)
