# Supplementary material for: Definition of the zebrafish genome using flow cytometry and cytogenetic mapping
Source: BMC Genomics. 2007 Jun 27;8:195. doi: 10.1186/1471-2164-8-195 (PMC1925092; doi:10.1186/1471-2164-8-195)
Supplement: Additional file 3 — A table listing the BAC clones observed to hybridize to multiple locations within the zebrafish genome using FISH. [file 1471-2164-8-195-S3.pdf]

Additional file 3. A table listing the BAC clones observed to hybridize to multiple locations within the zebrafish genome using FISH.

BAC clone	LG by UCSC genome browser*	LG by Ensembl (Zv6-version 40)*	LG by Sanger WebFPC database*	LG by Vega (v20)*	Number of chromosomes with signals
zC034K01	15	15	15	15	2
zC056F12	3 and 16	3	3	NA	2
zC059G18	9 and 20	9, 12, and 14	14	NA	25
zC106F05	15	15	15	NA	2
zC113C15	U†	NA	18	NA	2
zC127B06	12	20	15	15	4
zC140G02	18	NA	18	NA	2
zC141M16	U	13 and 14	13	13	2
zC154A22	U	15	15	15	2
zC165K16	18	18	18	18	2
zC211K08	22	22	22	22	2
zC213I06	U	6 and 21	6	6	2
zC214J02	7	7	7	7	3
zC266I02	14	14	13	13	2
zC270H11	NA	NA	10	10	>5
zC273A05	5	6 and 14	14	14	25
zC273E20	NA	NA	14	NA	2
zK006A12	18	NA	18	NA	>5
zK011B07	11	NA	NA	NA	25
zK023F09	11	NA	11	NA	2
zK024H22	10	NA	1	1	2
zK048M05	10 and 17	NA	10	NA	4
zK075B12	14	NA	NA	NA	2
zK110G07	3	NA	3	3	2
zK110G19	13	NA	4	4	1 (2 signals)
zK120I24	U	NA	NA	U	>5
zK168J09	22	NA	22	22	>5
zK171K22	7	NA	7	NA	25
zK171O17	22	NA	22	22	>5
zK173B08	2	NA	NA	NA	25
zK246K02	14	NA	14	14	2

*Based upon data obtained as of August 2006

†Mapped unambiguously to an unknown chromosome (chromosome U)

‡NA: BAC clone is not in the database
