# Supplementary material for: Definition of the zebrafish genome using flow cytometry and cytogenetic mapping
Source: BMC Genomics. 2007 Jun 27;8:195. doi: 10.1186/1471-2164-8-195 (PMC1925092; doi:10.1186/1471-2164-8-195)
Supplement: Additional file 4 — A table summarizing the sequencing status of the 510 BAC clones cytogenetically mapped to unique chromosomal locations in this study. [file 1471-2164-8-195-S4.pdf]

Additional file 4. A table summarizing the sequencing status of the 510 BAC clones cytogenetically mapped to unique chromosomal locations in this study.

	No. of clones*	Percent of clones
Sequencing complete and match	441	86.5
Completely sequenced and 2 BAC ends sequenced	147	33.3
Complete sequence only	19	4.3
2 BAC ends only	251	56.9
1 BAC end only	24	5.5
No significant matches	13	2.5
Sequencing in progress	54	10.6
Sequencing cancelled	2	0.4

*Total number of clones based upon the 510 BAC clones assigned to a unique LG chromosome by FISH mapping
